# Reducing language to rhythm: Amazonian Bora drummed language exploits speech rhythm for long-distance communication

**DOI:** 10.1098/rsos.170354

**Published:** 2018-04-25

**Authors:** Frank Seifart, Julien Meyer, Sven Grawunder, Laure Dentel

**Affiliations:** 1 Amsterdam Center for Language and Communication, University of Amsterdam, 1012 CX Amsterdam, The Netherlands; 2 Department of Linguistics, University of Cologne, 50923 Köln, Germany; 3 Université Grenoble Alpes, CNRS, GIPSA-lab, 38000 Grenoble, France; 4 Linguistics Division, Museu Paraense Emilio Goeldi, Belém, Brazil; 5 Department of Human Behavior, Ecology and Culture, Max Planck Institute for Evolutionary Anthropology, 04103 Leipzig, Germany; 6 Department of Linguistics, Kiel University, 24118 Kiel, Germany; 7 The World Whistles Research Association, Paris, France

**Keywords:** drummed speech, speech rhythm, Bora language, Amazonia, talking drums, intervocalic duration

## Abstract

Many drum communication systems around the world transmit information by emulating tonal and rhythmic patterns of spoken languages in sequences of drumbeats. Their rhythmic characteristics, in particular, have not been systematically studied so far, although understanding them represents a rare occasion for providing an original insight into the basic units of speech rhythm as selected by natural speech practices directly based on beats. Here, we analyse a corpus of Bora drum communication from the northwest Amazon, which is nowadays endangered with extinction. We show that four rhythmic units are encoded in the length of pauses between beats. We argue that these units correspond to vowel-to-vowel intervals with different numbers of consonants and vowel lengths. By contrast, aligning beats with syllables, mora or only vowel length yields inconsistent results. Moreover, we also show that Bora drummed messages conventionally select rhythmically distinct markers to further distinguish words. The two phonological tones represented in drummed speech encode only few lexical contrasts. Rhythm thus appears to crucially contribute to the intelligibility of drummed Bora. Our study provides novel evidence for the role of rhythmic structures composed of vowel-to-vowel intervals in the complex puzzle concerning the redundancy and distinctiveness of acoustic features embedded in speech.

## 1. Introduction

The human voice can produce rich and varied acoustic signals for transmitting information, but normally only up to a few hundred metres. Drum communication systems like that of the Bora people of the northwest Amazon can extend this range by a factor of up to 100 through emulating speech in sequences of drumbeats. But using acoustically simple signals that propagate well in natural environments across long distances comes with the challenge of being able to represent only a fraction of the acoustic features of spoken language, as produced by the human voice, without rendering speech incomprehensible [1]. Systems of long-distance communication emulating speech with drummed beats or whistles developed by traditional societies thus represent unique natural laboratories for exploring the relative distinctiveness of various linguistically relevant acoustic features. Although the importance of speech emulation for a better understanding of the architecture of human language has long been recognized [2], existing descriptions of drummed speech are still largely impressionistic and lack the methodological rigour of modern phonetics. Previous studies have highlighted two features of drummed speech: (i) ‘enphrasing’, i.e. elaborating words and sentences to make them longer and less ambiguous, and (ii) the representation of linguistic tone [3–11], but have almost entirely neglected exploring the role of speech rhythm, despite the clearly rhythmic nature of drumming. For Bora, it has even been claimed that rhythm plays no role in drummed speech [12], wrongly as we will argue.

The rhythmic organization of speech is complex and an area of extensive experimental research and lively theoretical debate [13]. Currently debated issues include what should be identified as the carrier of rhythmic beat in spoken languages [14], whether vowel-to-vowel intervals represent basic units of speech rhythm [15] and what is the role of rhythmic structures in language comprehension [16]. Against this background, we ask here which aspects of rhythmic structure are selected in conventionalized drummed speech to optimally contribute to its intelligibility.

To address this question, we carried out a case study on the Bora drum communication system from the northwest Amazon, locally known as *manguaré*, investigating in detail the prosodic features in common between spoken and drummed speech. Drummed Bora represents Bora words with a number of drumbeats that equals the number of its syllables. We focus on word-internal rhythmic units delimited by beats and especially their durations, as explained later in this Introduction. We analyse a corpus of drummed Bora messages showing how drummed speech selects and combines tonal and rhythmic features of the Bora language to communicate a wide variety of messages. In addition to an experimental study on rhythm, we carried out qualitative analyses of tonal, lexical and grammatical properties of drummed and spoken Bora. Taken together, these constitute the most comprehensive linguistic analysis of a drum communication system so far. The present study is part of a larger project that involved an extensive multimedia documentation of this traditional drummed practice and spoken Bora [17], and a methodology to analyse drummed speech that was developed based on a smaller set of *manguaré* data [18].

### 1.1. Emulated speech systems and drummed speech

Drummed speech systems are attested in Africa, South America, Asia and Oceania [3,4]. They are one type of emulated speech system, all of which operate by transforming spoken language as produced by the human voice into sounds such as whistles or acoustic signals produced with musical instruments. Such systems have also been called ‘speech surrogates’ [4] or ‘speech substitutes’ [19] in the older literature. Of these techniques, drummed speech can not only reach the longest distances but also employs the most radical reduction in acoustic complexity in all three main dimensions of acoustic signals: frequency, amplitude and time. By comparison, whistled speech consists of a much less radical acoustic reduction, primarily in the frequency domain, and can still represent, for example, vowel qualities [1,20].

Drummed signals exploit the natural bio-acoustic properties of percussions for optimal sound propagation in natural environments [21]. Their low pitch frequencies are not blocked by large vegetation [22,23] and their high amplitudes and narrow frequency band reduce further noise-masking effects [24,25].

Drummed emulations of speech should be distinguished from various other types of drummed signalling systems. Firstly, there are systems that use repertoires of drummed codes to symbolically represent meaning, with no iconic relationship to the sound of a spoken language, as often found in Melanesia [26,27]. Secondly, there are musical drumming traditions that use nonsense syllables (so-called vocables) that iconically represent musical sounds for mnemotechnical purposes, but never carry linguistic meaning, as in the North Indian Tabla tradition [28]. By comparison, a purely symbolic association is also made in the occidental solfège to represent notes of the scale by the syllables *do*, *re*, *mi*, *fa*, *sol*, *la*, *ti*. Finally, drummed emulations of speech are also different from systems like the Morse code, which symbolically represents units of a writing system, and does not iconically represent speech sounds.

### 1.2. Tone, rhythm and enphrasing in drummed speech

The most comprehensive review on drummed speech [4] provides information on 18 different forms of drummed speech, mostly by reprinting older descriptions up to the first half of the twentieth century, on e.g. Ewe, Twi/Akan, Banen (all from West Africa), Chin (from Burma) and an early, short report on Bora [5]. From these descriptions and the few published since on this subject (e.g. [29]), it emerges that such systems most frequently represent two acoustic properties of spoken language: tonal contrasts and rhythmic structures. In fact, drummed speech has been attested almost exclusively for tonal languages, although one rare exception has recently been described [6,30,31]. In tonal languages, drummed speech systems render abstract, underlying phonological tone categories through different drum pitches. Level tones, e.g. high versus low, are represented by single beats on drums with different pitches, and contour tones, i.e. rising or falling, by a succession of two beats of different pitches.

The representation of rhythm in drummed speech has barely been addressed in previous research, beyond the observation that beats correspond to syllables and that longer pauses occur at boundaries between phrases or sentences [7,8]. As far as we know, only two previous studies have analysed rhythmic aspects of drummed speech in some detail: Nketia [9] found that in Akan (Ghana) drummed speech, closed syllables are represented by longer pauses between beats than open syllables followed by a voiced consonant or just a vowel. More recently, Cloarec-Heiss [10] argued that for drummed Banda-Linda (Central African Republic), the liquid consonants [l, r] and the labial fricative [v] are represented by shorter intervals between beats than other consonants in drummed speech. However, both of these studies remain impressionistic in that they do not provide any quantitative phonetic measurements.

Finally, it has been noted that drummed speech has an intrinsically limited semiotic potential due to the reduced acoustic distinctiveness of the drummed signal when compared with human speech. This leads to ‘drum homophones’, i.e. different spoken words that sound the same in the drummed mode [32]. As far as we know, all drummed speech systems counter this by ‘enphrasing’, i.e. short words that would come out as homophones in drumming are replaced by longer, less ambiguous expressions [4], often with poetic creativity [33].

### 1.3. Speech rhythm

Durational patterns contribute to shaping speech rhythm even in languages where contrastive duration is not employed to signal phonological oppositions, e.g. vowel length. Perceptually, rhythm appears to be built on temporal structures that rely on longer intervals, as opposed to shorter ones, and the expectation of regular patterns by listeners results in perceiving sequential events as being grouped together into higher-level patterns [34]. Such grouping is common to both language and music and constitutes an essential step in the interpretation of complex sound sequences. Temporal structure refers to the durational patterning of events, which is measured by the time intervals between successive events. The rhythmic structure of speech involves a temporal organization into intervals of various types, such as syllables or stress [28], which may be emulated in drummed speech. The duration of such intervals and their potentially hierarchical organization are determined by a complex set of factors, some of them language-specific [13]. To investigate speech rhythm in the sense of a sequence of intervals with (potentially) different durations, measurements of syllable (as well as phoneme) durations have been carried out on a range of languages with the aim of characterizing languages as a whole as belonging to distinct rhythmic types [35] as well as to characterize individual speakers [36]. Measures for this purpose include the standard deviation of the duration of vocalic (ΔV) and consonantal (ΔC) intervals, the percentage to which speech is vocalic (%V) [37] and the pairwise variability index [38], a measure of the degree to which adjacent syllables or vocalic/consonantal intervals contrast in duration. This research revealed, firstly, that languages systematically differ with respect to durational contrasts between syllables, as well as between consonantal and vocalic segments of the syllables. Results of such measurements have also been used to investigate whether languages tend to approximate rhythmic isochrony for syllable durations or inter-stress interval durations [39,40]. But, this classification remains contested [41,42]. Secondly, it was shown that speakers from a very early age on can discriminate languages based on low-pass filtered speech samples preserving rhythmic properties [43].

Rhythmic units associated with syllables also play an important role in grammar in the sense that many languages categorize syllables, treating one class as heavier than another, for purposes such as stress placement and poetic rhyme [15]. Such weight distinctions usually, but not always, correlate with phonetic duration [44]. Syllable weight is often binary and described in terms of moras (light syllables: mono-moraic; heavy syllables: bi-moraic). The moraic model must assume that the weight of coda consonants is subject to language-specific parametrization representations designed to account for languages that treat both CVV and CVC as heavy, while others treat only a subset of these syllable types (usually CVV) as heavy. Note also that consonants in syllable onsets do not contribute to weight in terms of moras [44]. However, the concept of mora cannot easily cope with the fact that weight hierarchies can be highly complex, beyond two or three levels, and that onsets may, in fact, contribute to syllable weight [45], a famous example being C_voiced_ V* < *C_voiceless_ V* < *VV *< *C_voiced_ VV *< *C_voiceless_ VV in Pirahã [46].

It has recently been proposed that the units of speech rhythm that encode weight for the purpose of stress placement might be vowel-to-vowel intervals (delimited by the beginning of each vowel, see further discussion and illustration below) rather than syllables, so far with mixed results [15,47,48]. In any case, weight distinctions of vowel-to-vowel intervals also correlate with phonetic duration [48]. This proposal is anchored in the strong contribution of the vowel onset as an acoustic correlate for the perception of the beginning of speech events [49,50]. Other studies suggest that the relevant rhythmic units are most accurately delimited by the p-centre [51], roughly, the perceptual ‘beat’ of the syllable, which approximates the beginning of the vowel but anticipates it slightly for longer onsets [52]. This is supported by findings showing that syllable onsets do contribute to syllable weight, but less so than codas, for the perception of syllable duration and for stress placement [51,53]. Accordingly, probabilistic scales of weight differences between rhythmic units (as a function of onset length) have been proposed that successfully predict stress placement [51].

From the literature reviewed above, we know that speakers are sensitive to fine-grained durational contrasts of intervals associated with syllables for language discrimination and stress placement. We also know that rhythmicity of speech contributes to intelligibility by allowing for predictability of important information contained in syllable nuclei or stressed syllables [54]. However, it has hardly if ever been directly investigated so far to what extent durational contrast of rhythmic units might enable speakers to link acoustic input to meaningful elements, i.e. words and phrases, and if so, which kind of units can perform this function. It is likely that durational contrasts of rhythmic units can fulfil this function for two reasons. Firstly, many languages employ durational contrasts at the level of consonant and vowel phonemes. For instance, two length contrasts in vowels are common [55], but three are also well attested [56]. Secondly, experimentally reduced speech (noise-band vocoding, sine-wave speech and acoustic chimaeras) remains highly intelligible after removing acoustic cues distinguishing consonants and vowels but preserving temporal cues, including the rhythmic contours resulting from the frequency and amplitude pattern of the resonance peaks of a natural utterance [16,57–59].

### 1.4. Bora *manguaré* drumming

The Boras are an indigenous group of approximately 1500 members residing traditionally in small communities centred around multi-family roundhouses in the Amazonian rainforest of Colombia and Peru [12,60]. *Manguaré* drums are an important element of Bora culture [61] and were traditionally present in every roundhouse. They are used for communication within and across communities, up to 20 km away. Bora *manguaré* is one of the very few ancient drum communication systems attested in South America [62], where it developed independently of potential influence from Africa through the colonial slave trade [63]. In principle, every adult Bora man can use the drums and every community member can understand *manguaré* messages, both of which are acquired without explicit training. Sources from the early twentieth century report that *manguarés* were in daily use at the time, including for conversations about almost any subject and that messages were relayed from one roundhouse to another to reach further distances [64]. Today, the approximately 20 *manguaré* drums still in existence among the Boras are gradually falling out of use, just as spoken Bora is giving way to local Spanish.

**Figure 1.**
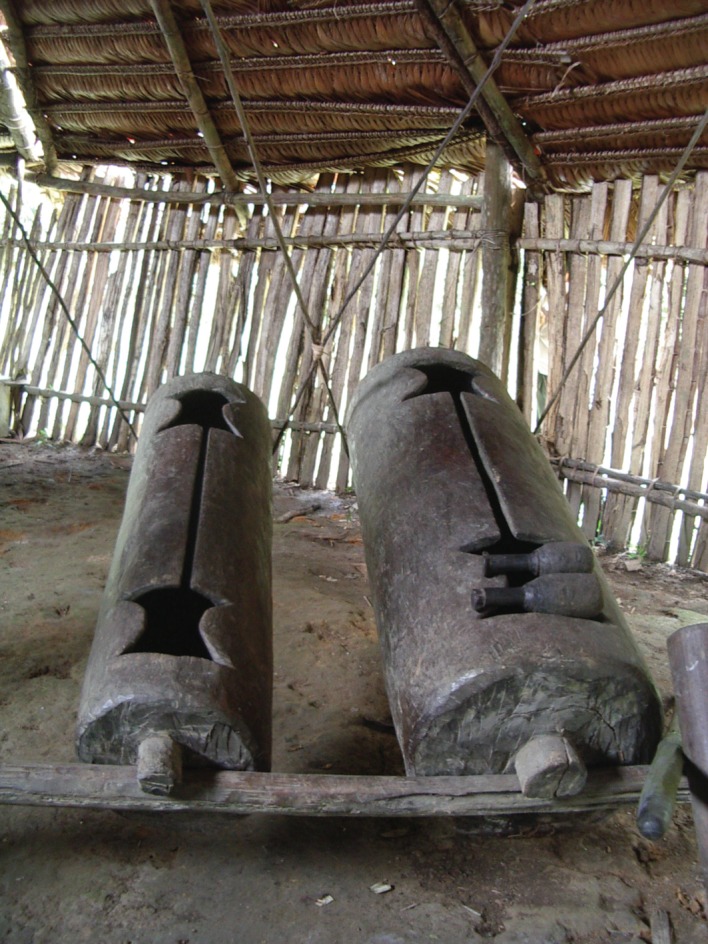
*Manguaré* drum pair in Brillo Nuevo, Peru (Photo Julien Meyer/Laure Dentel).

**Figure 2.**
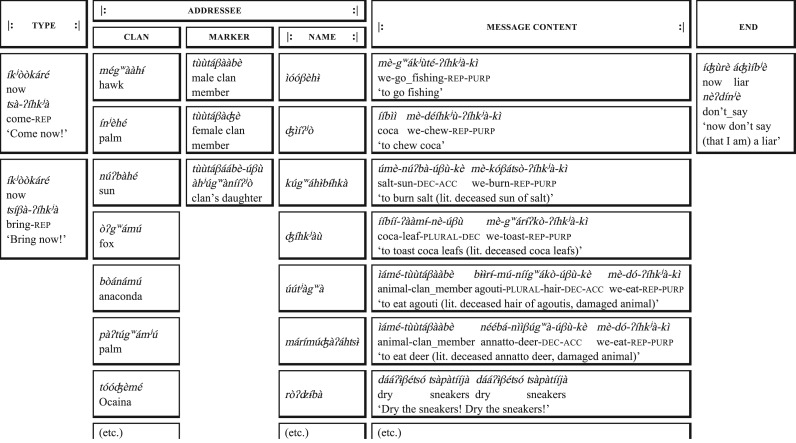
The structure of *manguaré* ‘calling messages’. Segmentation into meaningful elements and translation of each of them, in addition to idiomatic translations, are provided. Abbreviations: rep: repetitive; purp: purposive; dec: deceased; acc: accusative case.

**Figure 3.**
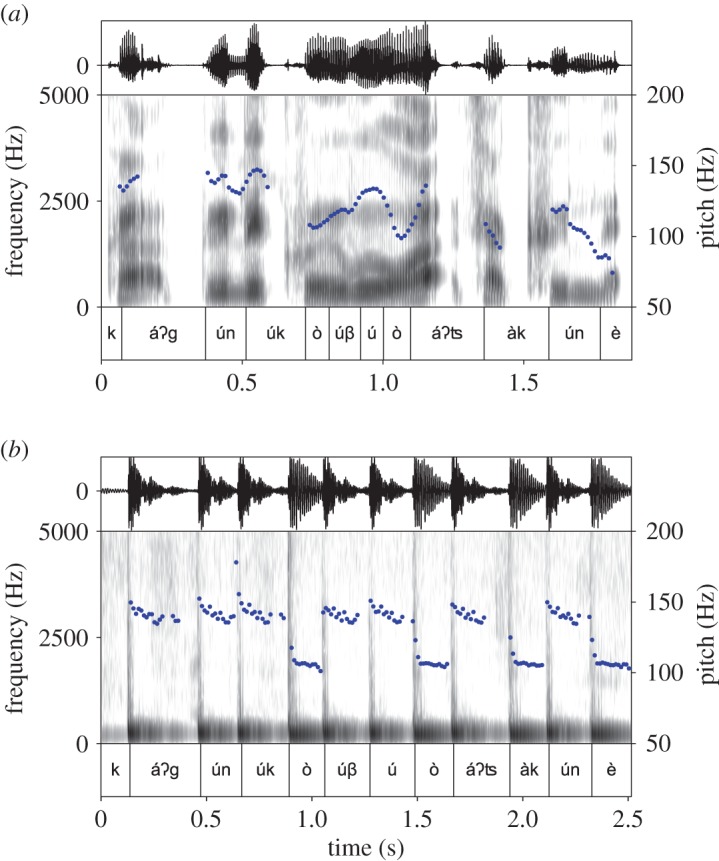
Acoustic properties of the Bora phrase *káʔgúnúkòúβú ò áʔʦàkúnè* ‘I am finishing the cahuana (manioc starch drink)’, in spoken (*a*) and drummed (*b*) versions. Oscillograms (top rows of *a* and *b*) represent acoustic energy as sound pressure over time. Spectrograms (middle rows) represent the spectrum of frequencies that is interpretable as linguistically distinctive acoustic information. Blue dotted lines indicate pitch. Bottom rows provide transcription and segmentation into vowel-to-vowel intervals (see the text for explanation). Figure created in PRAAT [65]. Corresponding sound files are provided in electronic supplementary material, S3 and S4.

Bora *manguaré* drums are pairs of wooden slit drums carved from single logs (traditionally by burning, due to lack of metal instruments), each approximately 2 m long (figure 1). The drums are hung in parallel in a wooden structure and the drummer stands in the middle to beat them with two wooden mallets covered with natural rubber. Each drum can produce two pitches (one on each side of the slit) and therefore the pair of drums can produce four pitches. The smaller drum produces higher pitches than the larger one. Not all uses of *manguaré* drums iconically emulate speech. Two uses can be identified in Bora [12] and characterized in terms of an earlier description of Akan drummed speech [9]:

1. The ‘musical mode’ of *manguaré* is used to perform memorized drum sequences with no or very little variation as part of rituals and festivals. These melodic, musical sequences make use of all four pitches. A subtype of ‘musical mode’ sequences are associated with syllables of sung lyrics (the ‘singing mode’). Other sequences do not correspond to syllables of spoken languages at all, like instrumental music without lyrics. In the ‘musical mode’, drumbeats follow a fixed, isochronous rhythm.
2. The ‘talking mode’ of *manguaré* is used to transmit relatively informal messages and public announcements, e.g. to ask someone to bring something or to come to do something (figure 2), to announce the outcome of non-alcoholic drinking competitions (figure 3) and to announce the arrival of visitors or the return of the chief of a roundhouse. In this mode, only two pitches (one of each drum) are used and each beat corresponds to a syllable of a corresponding phrase of spoken Bora. The informative nature of these messages puts pressure on both the emitter and the listener for an effective transmission of meaning and therefore the ‘talking mode’ is of most interest to the present study. Note that ‘talking mode’ messages can be preceded by ‘musical’ sequences, during which both drums may be played simultaneously, thus fulfilling the function of ringtones.

### 1.5. Bora phonology

Importantly for the study of *manguaré*, Bora is a two-tone language, i.e. every syllable is associated with either high (H, e.g. *bá*) or low (L, e.g. *bà*) tone [12], which are expressed by fundamental frequency pitch contours (figure 3*a*). Bora has six phonemic vowels, /*i*, 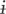, *u*, *e*, *a*, *o*/ (/*u*/ and /*e*/ are phonetically [*ɯ*] and [*ε*]). The Bora plain consonants /*p*, *b*, *t*, *d*, *ʦ*, *ʣ*, *ʧ*, *ʤ*, *k*, *g*, *g^w^*, *ʔ*, *β*, *r*, *m*, *n*, *h*/ almost all have palatal(ized) counterparts, e.g. /*p^j^*, *b^j^*, *t^j^* /. From now on, we will adopt the phonemic transcription without slashes (/). Bora has a (C)V(h/ʔ) syllable structure, i.e. an optional consonant (C) in the syllable onset, an obligatory vowel (V) and an optional consonant in syllable coda position, which can only be *h* or the glottal stop *ʔ*. Therefore, the only consonants preceding other consonants in Bora are the glottal fricative *h* and glottal stop *ʔ*, which also occur in other positions. This restricted distribution is due to a historical development from geminate consonants [66], but, synchronically, *h* and glottal stop *ʔ* behave like other consonants of the language. Additionally, Bora phonologically distinguishes short versus long vowels (written as sequences of two identical vowel symbols), but these long vowels cannot be followed by *h* or *ʔ* within one syllable. Bora phonology distinguishes light, i.e. mono-moraic (C)V syllables from heavy, i.e. bi-moraic (C)VC and (C)VV syllables for various phonotactic and morphophonological purposes [12].

### 1.6. Aims of this study

**Figure 4.**
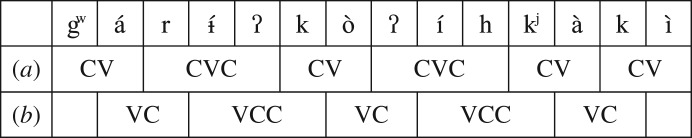
Alternative segmentations of Bora 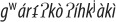 ‘in order to toast’ into (*a*) syllables and (*b*) V-to-V intervals. Note that word-initial consonants are associated with preceding V-to-V intervals and word-final vowels with the following ones within phrases.

To understand the role of rhythm in drummed Bora, we firstly tested which rhythmic units are represented in drummed Bora based on measurements taken in corpora of drummed and spoken Bora. Secondly, we carried out qualitative analyses of the tonal, lexical and grammatical aspects of spoken and drummed Bora that interact with rhythmic structure. Regarding the specific hypothesis tested in the experimental study, it is clear that in *manguaré* messages, the number of beats equals the number of syllables. However, this leaves open various possibilities regarding which consonants preceding or following a vowel are included in the rhythmic units that are represented as intervals between beats and thus determine drummed interbeat durations (IBDs). We test primarily the two main competing theories of rhythmic units, the syllable and the vowel-to-vowel (V-to-V) interval. Through testing the syllable hypothesis, we are also able to test other derived possibilities, namely the ‘vowel length-only’ hypothesis, derived from a remark in the earliest, brief description of Bora *manguaré* [5], and the ‘mora’ hypotheses, derived from Bora phonotactics. As mentioned above, V-to-V intervals are delimited by the beginnings of vowels (and so are vowel length-only intervals and mora intervals), i.e. the syllable nuclei that carry the peak of acoustic energy in both spoken language and drummed speech (figure 3). The syllable and V-to-V types of units correspond to different segmentations of all speech segments into rhythmic units. They yield the two following different associations between drummed IBDs and phonemes:

- *Syllable*: IBDs are associated with the content of corresponding syllables (figure 4, row (*a*)). Possible syllable types in Bora are V, CV, VC (Vh and Vʔ only), CVC (CVh and CVʔ only), VV and CVV (where VV represents long vowels). (Note that the vowel length categories V and VV as well as the moraic categories V and {VV,VC} can be derived from the syllable case.)
- *V-to-V interval*: IBDs are associated with the phonemes present between the beginning of one vowel up to the beginning of the following vowel (figure 4, row (*b*)). Possible V-to-V interval types in Bora are V, VC, VCC (VhC and VʔC only), VV and VVC. Note that VV intervals, i.e. long vowels followed directly by another vowel with no intervening consonant, are exceedingly rare in Bora. The V-to-V type VV did not occur at all in the drummed corpus and was very rare in the spoken corpus and could therefore not be tested.

We hypothesize, firstly, that Bora drumming represents V-to-V intervals, based on the iconic association of prominent acoustic peaks in drumming and spoken language, rather than syllables, only vowel length or only moras, and that these rhythmic units should be consistently respected across different drummers. Secondly, we hypothesize that the V-to-V intervals we identified in drumming should also be observable in spoken Bora. Thirdly, we hypothesize that phonological tones play a minor role in the intelligibility of drummed messages when compared with rhythmic units. Finally, we hypothesize that special lexical and grammatical devices are employed in drummed message to support the intelligibility of drummed Bora. Below, we address these hypotheses in roughly reverse order because lexical, grammatical and tonal characteristics provide necessary background for discussing the rhythmic properties.

## 2. Material and methods

### 2.1. Materials

The analysed data originate from an extensive corpus of *manguaré* and spoken Bora collected as part of a large-scale project documenting Bora linguistic and cultural heritage [17]. The corpus contains 169 *manguaré* drummed messages that were performed by a total of five native Bora speakers. These were recorded on video and non-compressed audio using a Sony ECM-MS907 microphone. Messages contain on average of 15 words and 60 drumbeats. The majority of the messages were systematically elicited on the basis of a survey of the types of drum messages that are in common use to ensure coverage of message types, typically occurring content and of different drummers. Also included are some messages that were performed spontaneously, e.g. when calling community members for a communal fishing event.

All drummed messages were transcribed, i.e. sequences of drumbeats were annotated with corresponding Bora phrases, and translated together with a native Bora speaker while doing on-site fieldwork using ELAN [67]. This ensured that all messages included in the analysis are interpretable for Bora speakers. We excluded from the analysis drummed data in which the number of drumbeats or their tone category did not match the intended Bora phrase due to ‘slips of the drum’ that were identified during fieldwork together with a native speaker (approx. 5% of the recorded messages). All data have been archived and are accessible online [17]. They represent, by far, the most comprehensive database accessible online of any drummed speech system so far.

We then selected a set of recordings of spoken Bora for comparison with Bora *manguaré*. These recordings are comparable with drummed messages in terms of, e.g. spontaneity, and were produced by the same Boras who contributed the drummed data. They include spoken versions of some of the phrases and personal names that typically occur in drummed messages, as well as recordings of Bora names of zoological species. These data were then analysed with the same methodology as for the drummed data.

### 2.2. Design and procedure

For the experimental study, each recorded drum message was analysed as a function of its associated phonemic content (vowels, consonants and phonological tone) as annotated during the field documentation. For each drumbeat, the pitch (high or low) and the exact time position (based on the point with the highest acoustic energy) were automatically identified using PRAAT [65]. This identification was manually double-checked by two researchers. Similarly, a measure point was manually set at the vowel onset in spoken data, again with double-checking. This measure point was set at the third cycle of oscillation of the vocalic segment, in accordance with phonetic standards [68]. In the drummed data, the H or L tone categories of the transcribed vowels matched with approximately 140 Hz (high-pitched drum) and 95 Hz (low-pitched drum), respectively, in the drummed signal, a difference which is easily perceptible by the human ear [21]. The duration of each interval was calculated from the time stamps and each interval was linked to the sequence of vowels and consonants of spoken Bora that it represents, as can be seen in figure 3.

For the analysis of the drummed corpus, we first selected the recordings of the drummer who had contributed the most data (our ‘first drummer’), who is also the one with the highest proficiency in drumming according to the Bora population in Peru. This subcorpus contains 2030 word tokens and 7459 intra-word intervals, i.e. pairs of drumbeats. To investigate consistency of the results across drummers, we selected the data of the drummer (our ‘second drummer’) who had contributed the second-largest amount of data in our corpus (1886 intra-word intervals). We identified a high rate of repetition in drummed messages, e.g. only 231 different word types in total in the subcorpus of the first drummer. The corpus of spoken Bora, produced by the same two drummers/speakers, consists of 557 word tokens with 147 different word types and 1278 intra-word intervals for the first drummer/speaker and 333 word tokens with 299 different word types and 731 intra-word intervals for the second drummer/speaker. To study some aspects of spoken–drummed correspondence in more detail, we identified 14 different word types that occurred in both our spoken and drummed corpora.

We statistically analysed the variations in the qualitative IBD variable as a function of several explanatory variables, including their interactions. Details of each model are provided in the next section. Additionally, durational differences between some classes of words were tested by applying non-parametric tests, because the normality assumption for a *t*-test was violated in these sub-samples. Details of these tests are provided in electronic supplementary material, S1-d.

## 3. Results and discussion

### 3.1. Grammar and lexicon of *manguaré* messages

In our analysis of the ‘talking mode’ of *manguaré* drumming, we found that messages follow a fairly rigid structure (or ‘syntax’), with a fixed order of building blocks of various types. The structure of the most frequent message type, ‘calling messages’, is represented in figure 2. Within this structure, the elements ‘(message) type’, ‘marker’ and ‘end’ in particular are more or less invariable, formulaic elements signalling the structure of a message. New information is principally communicated in the slots corresponding to the addressee's clan and proper name and especially in the ‘message content’, in which a potentially infinite number of different messages can occur.

We found that Bora *manguaré* messages apply three types of enphrasing devices, i.e. conventional ways of making words and phrases longer and less ambiguous. Firstly, with few exceptions, nouns and verbs are marked with special disyllabic markers. On nouns, the marker -*úβù* is used (which in spoken Bora is used on nouns referring to deceased humans), for instance in 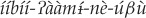 (coca-leaf-plural-deceased) ‘coca leaves’ (figure 2). For verbs, the marker -*ʔíhk^j^à* is used (which in spoken Bora signifies repeated action), for instance in *ʦíβà-ʔíhk^j^à* (bring-repetitive) ‘Bring!’ In drummed messages, these markers do not carry any semantic value, but function purely to identify the preceding sequences of beats as representing nouns or verbs. Such marking of grammatical information through enphrasing in drumming has, to our knowledge, so far not been described.

Secondly, the ‘marker’ element *tùùtáβà-àbè/-ʤè* (literally ‘the damaged one’) functions to identify preceding sequences as clan names and following sequences as proper names. Note that Bora speakers have no intuitions why elements with the literal meanings ‘deceased’, ‘repeated’ and ‘damaged’ should be used in *manguaré* message. This supports that they have been selected because of their rhythmic properties, as discussed below.

Thirdly, there are conventional long forms for words that occur frequently in *manguaré* messages to render them less ambiguous, as has been described for other drummed speech systems before [4,32]: for instance, the Bora noun referring to a commonly hunted deer species *nììβúg^w^à* is replaced in *manguaré* messages with *ìámé-tùùtáβààbè néébá-nììβúg^w^à-úβù*, literarily ‘deceased annatto deer, damaged animal’.

### 3.2. The representation of phonological tones

We found that, as expected, *manguaré* messages represent Bora high (H) and low (L) tones by beats on the small versus large drum, but not using the different tongues on each drum. The blue dotted lines representing pitch contours in figure 3*a* show that the realization of underlying tones in spoken Bora is modulated by an intonation contour with a characteristic ‘downdrift’ marking phrase boundaries. This information is not represented in drummed Bora, but only the two underlying tone categories, high and low (figure 3*b*). Note also that maximum amplitude levels of drumbeats are fairly constant and thus do not represent potentially informative amplitude variations of spoken language. Drummed high and low tones do display two different types of amplitude envelopes, namely a more rapid amplitude decline for high tone beats. But, this is intrinsically linked to the pitch properties of the smaller and less resonating hollow log used to produce high tone beats, which cannot be varied.

Our analysis of spoken Bora also confirmed that tone patterns carry primarily grammatical information, e.g. nominalization and phrase boundaries, and only very rarely differentiate words with different lexical meanings from each other. In addition, the occurrence of tone patterns is highly restricted by phonotactic rules which (i) prohibit sequences of two low tones except at the end of tone phrases (which may encompass various words) and (ii) impose word-final high tone in phrase-medial position and word-final low tone in phrase-final position [12]. Consequently, a trisyllabic Bora word can typically only have three out of eight logically possible tone patterns: HHH, HLH and LHH phrase-medially and HLL, HHL and LHL phrase-finally, i.e. a sequence of three tones in a given position in a phrase may represent any one out of one-third of all trisyllabic Bora words. For decoding drum messages, tone patterns thus facilitate parsing by indicating phrase boundaries and possibly encoding some other grammatical meanings. But for communicating lexical content, the distinctiveness of tone patterns is very limited.

### 3.3. Rhythmic structure

**Figure 5.**
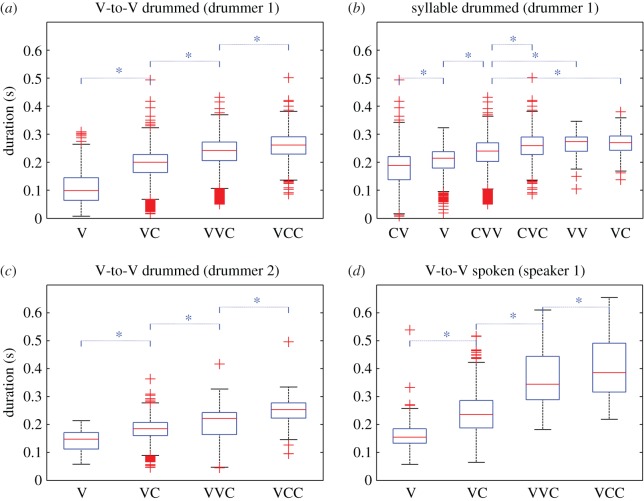
Distribution of drummed IBDs as a function of V-to-V types (*a*) and syllable types (*b*) for the first drummer, drummed IBDs as a function of V-to-V types for the second drummer (*c*) and durations of spoken V-to-V types (*d*) (VV, long vowel; CC, hC or ʔC). Further results are provided in electronic supplementary material, S1.

To find out which rhythmic units are represented in drummed Bora interbeat durations, we evaluated statistically which kind of hypothesis (syllable versus V-to-V; figure 5) explains best the IBD temporal distribution by constructing two different linear models of IBD (models 1 and 2, see tables 1 and 2) in R [69] and using the *lm* function to perform linear analyses. In each of these models, we included two variables as fixed factors: the variable DRUMMER and alternatively the variables corresponding to each one of the studied hypothesis (SYLLABLETYPE, V-TO-VTYPE). As there were only two drummers in this study, we included the variable DRUMMER as a fixed effect [70]. Using nested modelling techniques and *F*-tests, we performed a backward selection and we verified for each model that both DRUMMER and SYLLABLE (in model 1) and both DRUMMER and V-TO-VTYPE (in model 2) and their interactions had a significant effect on the IBD variations. Visual inspection of residual plots of both models did not reveal any obvious deviations from homoscedasticity or normality, nor the presence of influential outliers. Next, we performed multiple comparisons. For each of the models for which it was needed, the multiple comparisons were done with Hothorn *et al.*'s method [71] using the function *glht* from the package *multcomp* of R. This method allows us to perform multiple comparisons of means after building appropriate matrices of contrasts (using the function *lsmeans* from the package *lsmeans*). This ensures that the overall type I error associated with the simultaneous decisions does not exceed the pre-specified significance level (here 0.05, as usual) by adjusting *p*-values [69,71,72]. Finally, we compared the models by using the adjusted *R*^2^ values obtained for each model, as they indicate the part of variation in the IBD variable that is explained by the explanatory variables of each model, taking into account their number of parameters.

**Table 1.** Results for predictors and interactions present in model 1 (SYLLABLETYPE (V, CV, CVV, CVC, VV, VC) for each DRUMMER (1 and 2)).

variable	d.f.	sum square	mean square	*F-*value	Pr(>*F*)
DRUMMER	1	0.1265	0.12647	40.2258	2.367 × 10^−10^
SYLLABLETYPE	5	8.4501	1.69002	537.5405	<2.2 × 10^−16^
DRUMMER:SYLLABLETYPE	5	0.1248	0.02497	7.9418	1.772 × 10^−7^

**Table 2.** Results for predictors and interactions present in model 2 (V-TO-VTYPE (V, VC, VVC, VCC) for each DRUMMER (1 and 2)).

variable	d.f.	sum square	mean square	*F*-value	Pr(>*F*)
DRUMMER	1	0.1249	0.1249	42.427	7.716 × 10 ^−11^
V-TO-VTYPE	3	10.2952	3.4317	1165.560	<2.2 × 10^−16^
DRUMMER:V-TO-VTYPE	3	0.1763	0.0588	19.964	6.801 × 10^−13^

In the model testing the syllabic segmentation, the factors were SYLLABLETYPE (six levels: CV, VC, CVC, CVV, V and VV) and DRUMMER (two levels, 1 and 2). Nested modelling tests lead us to keep the interaction of second order between SYLLABLETYPE and DRUMMER (*F*_59 326_ = 7.94, *p *< 0.001). The adjusted *R*^2^ value was 0.2279.

Multiple comparisons between different levels of SYLLABLETYPE (see electronic supplementary material, table S1-4) showed that V, CV and CVV were significantly different in duration, while VV, VC and CVC were not significantly different from each other, although these three types were all significantly longer than V, CV and CVV (figure 5). As a consequence, the IBD scale for syllable types is assumed as follows: CV < V < CVV < {CVC, VV, VC}, with undetermined relative durations of the last three. While also distinguishing a total of four types of durational units, this scale does not match our expectations regarding the representation of consonants and vowels. In particular, consonants in onsets of syllables influence IBDs in a non-expected way: CV is significantly shorter than V, and CVV shorter than VV. In both of these cases, this would mean that withdrawing an onset consonant would add more duration to the syllable. An additional consequence of this syllable hypothesis is that drummed CVV syllables would be shorter than VC syllables, which is unexpected [73,74].

The IBD scale for syllable types suggests that heavy, that is, bi-moraic (C)VV and (C)VC syllables always correspond to longer IBDs when compared with light mono-moraic (C)V syllables. However, among the 30 comparisons between different syllable types (see electronic supplementary material, table S1-4), seven are not coherent with the hypothesis that only the contrast between mono-moraic and bi-moraic syllables would be transposed by the drums in IBDs. For instance, VC and VV syllables (both bi-moraic) are significantly different but should not be according to this hypothesis, just as CVV and VV syllables. In data from drummer 2, mono-moraic V syllables are not different from bi-moraic VV syllables. As a consequence, the binary opposition mono-moraic (C)V versus bi-moraic (C)VV and (C)VC syllables disregards the contrast between VC and VV sequences that we find in IBDs, as well as the contribution of syllable onsets to the duration of the preceding interval.

The hypothesis that only phonological vowel length is represented in drummed IBDs could also be tested using these results. By exploring the multiple comparisons between different levels of SYLLABLETYPE (see electronic supplementary material, table S1-4), we found that among the 30 comparisons, 21 are not consistent with this hypothesis. Some are inconsistent because the intervals compared should be different according to this hypothesis (e.g. VC versus VV) but are not, others because they are different but should not be (e.g. V versus VC) and yet others because they display an effect in the unexpected direction (V is longer than VV).

For testing the V-to-V hypothesis, the variables were V-TO-VTYPE (four levels: V, VC, VCC and VVC) and DRUMMER (two levels: 1 and 2). We added in the initial model their interaction of second order. The nested modelling test led us to keep the interaction between V-TO-VTYPE and DRUMMER (*F*_3,9336_ = 19.97, *p *< 0.001). The adjusted *R*^2^ value was 0.2777.

Multiple comparisons between different levels of V-TO-VTYPE showed that differences between all categories were statistically significant for both drummers (see figure 5 and the electronic supplementary material, table S1-5) resulting in a scale of IBDs as follows: V < VC < VVC < VCC. This scale reflects effects of both vowel length (VVC longer than VC) and of the number of consonants (VCC longer than VC and VC longer than V).

Taken together, these findings constitute evidence that the rhythmic units encoded in drummed Bora correspond more closely to V-to-V intervals (as annotated in figure 3) rather than to syllables, vowel lengths or moras. They show that consonants preceding a vowel are irrelevant for the IBD of the rhythmic unit associated with this vowel, while the number of consonants (including consonants that syllabify with following syllables) and the vowel length within V-to-V intervals are associated with significantly different IBDs, which are ordered in a coherent scale.

### 3.4. Consistency across drummers

Each Bora drummer has his own drumming style and rate, which may depend on factors such as arm length and body height. Therefore, we analysed data from different drummers separately. Comparison of results from both drummers shows that IBDs are consistently represented across drummers, except in one case for the syllable hypothesis. This confirms that rhythmic structure is represented consistently in drumming, and even more so for the V-to-V hypothesis of drummed Bora. (See data from the second drummer in figure 5*c* for V-to-V types and electronic supplementary material, S1 for syllable types.) Multiple comparisons showed that the same four V-to-V types were significantly different across drummers, and therefore that these followed the same scale, despite clearly observable individual differences (compare figure 5*a* with figure 5*c*). For syllable segmentation, the data of the second drummer displayed the same relative distribution and inconsistencies as for drummer 1, except that in data from drummer 2 V and CVV categories were not statistically different (see electronic supplementary material, figure S1-1 and table S1-4).

### 3.5. Rhythmic disambiguation in drumming

The standard procedure in linguistics to prove the distinctiveness of a phonetic feature is the identification of so-called minimal pairs, i.e. words that are distinguished only by this single feature. For example, *pet* versus *bet* proves that voicing of the consonants *b* versus *p* is distinctive in English because these two words have separate meanings. Following this procedure, we found many examples that prove that rhythmic structure is distinctive in drummed Bora. For instance, the three proper names *(ʤ)ì.íʔ^j^.ò*, *(n)èèp.áh^j^.ù* and 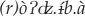 are all trisyllabic and have the tone pattern LHL. However, they contrast in the first V-to-V interval type they contain, V (*.ì.*) versus VVC (*.èèp.*) versus VCC (*.òʔʣ.*), which allows Boras to identify these names in drummed messages. Note that proper names occur in the same slot in the message scheme (figure 2) and thus there is no contextual information for further disambiguation except for the preceding clan name, which narrows down possible proper names. The distinctiveness of rhythmic structures compared to tonal patterns can be illustrated with the set of 18 trisyllabic proper names that occur in our drummed data (see electronic supplementary material, S1-c). In this set, seven rhythmically distinct sequences of intervals occur (e.g. V.VC.V versus VC.VCC.V), narrowing down the choice to on average 2.5 intended proper names. By contrast, only two distinct tonal patterns occur in this set, corresponding to on average nine distinct proper names each.

Quasi-experimental situations during transcription of drummed data, when short sequences of beats were presented in isolation, further show that Bora listeners reliably differentiate elements that are distinguished only by minimal rhythmic contrasts. An example here is *mèg^w^ák^j^ùtéʔíhk^j^àkì* ‘to go fishing’ versus *mèkóóβàtéʔíhk^j^àkì* ‘to bring fire wood’, which is tonally identical but rhythmically differentiated by the second interval (*ák^j^* VC versus *óóβ* VVC).

Maybe the most striking examples of rhythmic contrasts are the special markers for verbs (-*ʔíhk^j^à/á*) and nouns (-*úβù/ú*). As they have identical tone patterns (HH phrase-medially and HL phrase-finally), they must have been selected in the development of drummed Bora because of the distinctiveness of the rhythmic interval types involved, namely V (preceding -*úβù/ú*) versus VC (preceding -*(ʔ)íhk^j^à/á*) and VCC (*íhk^j^*) versus VC (*úβ*). Both of these interval pairs are indeed significantly different in mean duration in our data (see electronic supplementary material, S1-d).

Finally, Bora ‘drum homophones’ (i.e. words with identical tone patterns and rhythmic structures) that appear in similar contexts are conventionally elaborated in ‘enphrasing’. For instance, names referring to the commonly hunted animals 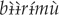 ‘agouti’ and *nììβúg^w^à ‘*deer’ (both LHL and (C).VVC.VC.V) are replaced in Bora drummed messages with longer expressions that include contrastive rhythmic structures and tone patterns (figure 2).

### 3.6. Comparison to spoken Bora

The tone patterns of drummed Bora represent underlying, abstract tone categories, which clearly have categorical status in the grammatical system of the language [12]. Our results on durational drummed interval types suggest that these similarly may represent abstract units, defined by different numbers of consonants and vowel length. But unlike for tones, the structural status of these intervals in the language is less clear. For instance, they are not relevant units for phonotactic rules, which are determined by syllables. To find out whether they have phonetic reality in spoken Bora, we applied the same analysis as for drummed Bora (see figure 5*d* for speaker 1 and electronic supplementary material, figure S1-1 for speaker 2). For this verification of V-to-V type for both spoken and drummed speech and their interactions (model 3, table 3), the variables were DRUMMER (two levels: 1 and 2), SPEECHTYPE (two levels: Drummed (D) and Oral (O)) and V-to-VTYPE (four levels: V, VC, VVC and VCC). The nested modelling test led us to keep the interaction between V-TO-VTYPE, DRUMMER and SPEECHTYPE (*F*_3,11 305_ = 3.52, *p* = 0.0142). Multiple comparisons between different levels of V-TO-VTYPE showed that differences in durations between all four categories were statistically significant for both drummers/speakers also in spoken data (see electronic supplementary material, table S1-6) and that these followed the same scale of IBDs observed in drummed data.

**Table 3.** Results for predictors and interactions present in model 3: V-TO-VTYPE (V, VC, VVC, VCC) for each DRUMMER (1, 2) and SPEECHTYPE (drummed versus spoken).

variable	d.f.	sum square	mean square	*F-*value	Pr(>*F*)
SPEECHTYPE	1	8.247	8.2474	2365.8540	<2.2 × 10^−16^
DRUMMER	1	0.434	0.4336	124.3909	<2.2 × 10^−16^
V-TOVTYPE	3	18.550	6.1833	1773.7490	<2.2 × 10^−16^
SPEECHTYPE:DRUMMER	1	0.162	0.1621	46.5061	9.599 × 10^−12^
SPEECHTYPE:V-TOVTYPE	3	2.791	0.9305	266.9219	<2.2 × 10^−16^
DRUMMER:V-TO-VTYPE	3	0.150	0.0501	14.3702	2.424 × 10^−9^
SPEECHTYPE:DRUMMER:V-TO-VTYPE	3	0.037	0.0123	3.5294	0.0142

**Figure 6.**
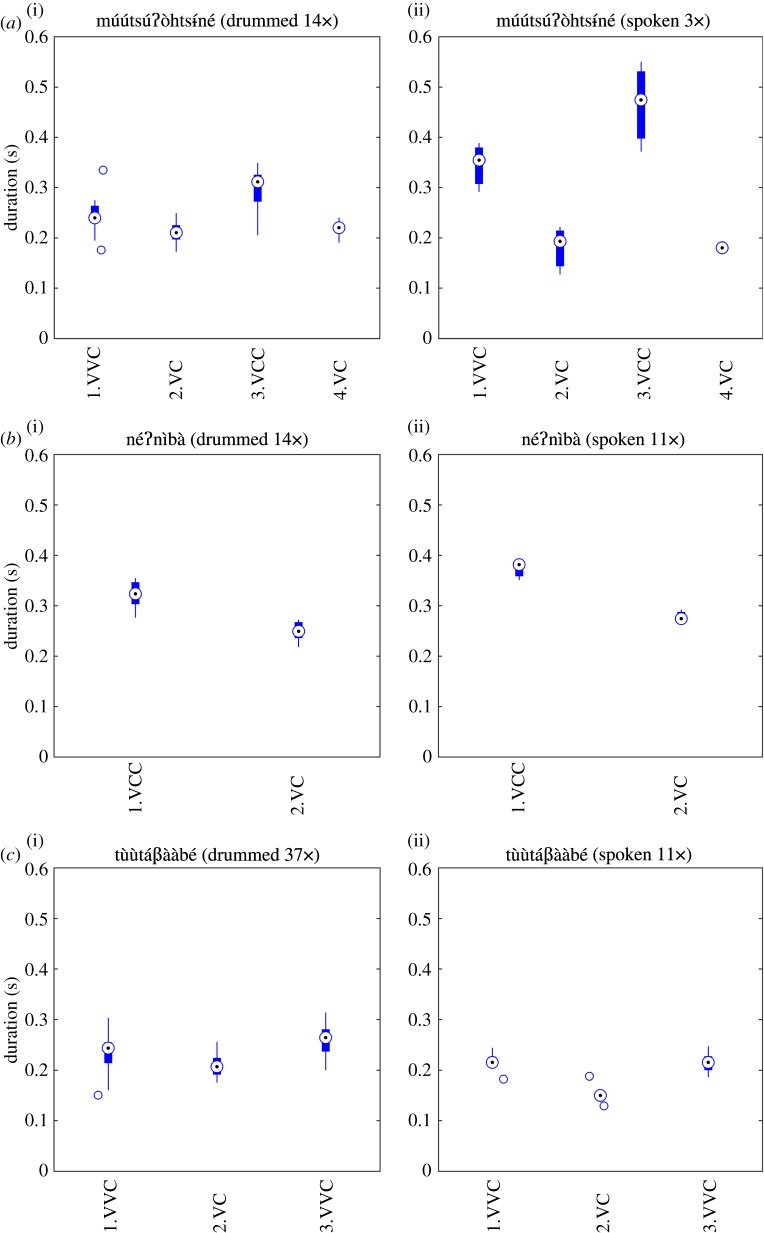
V-to-V durations as a function of V-to-V types for three Bora words (*a*–*c*) in their drummed (i) and spoken (ii) forms (VV: long vowel; CC: hC or ʔC).

Another way to approach this question is to compare rhythmic properties of words that occurred in both the spoken and the drummed corpora (14 items for the first speaker/drummer). Here, we found that the relative durations between consecutive V-to-V intervals in the drummed form always corresponded to those in the spoken form in each individual word. Results for three words are given in figure 6. For instance, the word 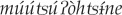 contains the V-to-V intervals *úúʦ* (VVC), *úʔ* (VC), *òhʦ* (VCC) and 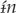 (VC), among which the expected relative durations VC < VVC < VCC were realized in both spoken and drummed forms.

We thus conclude that the rhythmic structure of drummed Bora not only represents abstract, underlying units but also matches surface realizations of spoken Bora. To distinguish four interval types, drummed Bora makes use of the possibility of gradual distinctions in interval length, as against the strictly two-way distinction in pitch that *manguaré* drums are restricted to.

## 4. Conclusion

Conventionalized systems for emulating speech using drums are natural speech registers that have developed independently across the world and are produced and comprehended with great ease. However, how exactly they encode speech has so far remained poorly understood and has led to various misconceptions [12]. Here, we show for drummed Bora the complex interplay between the representations of linguistic tone, speech rhythm and enphrasing, and highlight the crucial role of durational rhythmic units. Regarding enphrasing, we show that this is not only a device for the simple replacement of words for longer, less ambiguous expressions [32], but that it can also be used to mark grammatical categories such as nouns versus verbs.

Regarding tone, our current knowledge of systems emulating speech with drums suggests that such systems overwhelmingly develop in tone languages. Interestingly, Bora's neighbouring, non-tonal languages Ocaina and Witoto also use *manguaré* drums, but only for restricted, symbolic signalling or in a ‘musical mode’. This suggests that phonological tone may be a feature that strongly favours the development of such systems. However, our results suggest that representing tone is not sufficient for an efficient system with the expressive range of Bora *manguaré*, because tone patterns alone are not particularly distinctive in this language. This appears to be the case also in other languages with drum communication systems. The typology of linguistic tone suggests that in languages with two or three contrastive tones (as against four or more), tone tends to have a low functional load and rarely distinguishes lexical items [75], also in West African languages [76] for which drummed speech is attested.

Regarding rhythm, our study highlights the importance of non-isochronous rhythmic structure embedded in speech, as against isochronous rhythmic structures that aid parsing of utterances into words, syllables and phonemes [28,77,78]. We found that Bora *manguaré* exploits the semiotic potential of non-isochronous rhythmic structure in speech for linking acoustic input to meaningful elements, i.e. for word recognition, and is in line with previous experimental evidence for the actual exploitation of such dynamic temporal patterns in speech comprehension [16,58,79].

We resolved two crucial questions regarding the nature of such rhythmic patterns in speech, as represented in drumming. Firstly, we provided evidence that the rhythmic units encoded in drummed Bora match vowel-to-vowel (V-to-V) intervals more closely than syllables, i.e. that drumbeats align with vowel nuclei irrespective of onset consonants within the same syllable, which are associated with preceding intervals. The fact that drummed Bora, as a naturally evolved practice, selects to represent these units lends credibility to the concept of V-to-V intervals in the rhythmic structure of spoken languages. Previous support for vowel-to-vowel (V-to-V) intervals comes, for instance, from phonetic research on tonal alignment [80], on the importance of vowel onsets as an acoustic correlate of speech rhythm [50] and research dealing with syllable weight as a unit measure of quantity (e.g. [81]), but also from psycholinguistic studies on the notion of perceptual centre (p-centre)—the perceived moment of the occurrence of an acoustic event—which is commonly non-congruent with the physical signal onset [52,82] and is more closely aligned to the beginning of the vowel than the beginning of the syllable [51,83]. Moreover, the vowel-to-vowel interval appears to explain other aspects related to speech rhythm such as poetic rhyme in different languages and can possibly also account for stress placement [47].

Secondly, we found four distinct rhythmic units in data from two Bora speakers, both in spoken and drummed forms of speech, representing different numbers of consonants and vowel length: V < VC < VVC < VCC. As shown by our data, these units are differentiated by approximately 20 ms, which is within the resolution of differentiation for the human perceptual and cognitive systems for e.g. acoustic events for beats in experimental situations [84,85] and for phonetic events in spoken signals [25,53,86].

We thus submit that the representation of this kind of rhythmic structure plays a crucial role in drummed speech to reliably differentiate elements that are only distinguished by rhythmic structure, and to successfully communicate entirely novel and unexpected messages, e.g. the one in the bottom row of figure 2.

Language is a redundant system, which simultaneously provides many cues to enable the comprehension of the message encoded by the speaker, including contextual, grammatical and phonetic cues. For example, detailed qualitative properties of phonological segments are perceived and processed in language comprehension [87], such as the resonances differentiating vowels or the noise associated with fricative consonants, as visualized in the spectrogram in figure 3*a*. The current study shows that under certain circumstances, such qualitative distinctions can be entirely dispensed with. This is indicated by the empty spaces between beats in the spectrogram in figure 3*b*, where speech is reduced to rhythm combined with two pitch levels. To render drummed messages less ambiguous, drummed speech systems then elaborate lexicon, morphology and syntax. Such naturally evolved systems thus provide living models for a deeper understanding of the complexity of human language, regarding key questions in language sciences such as the interaction of redundancy, reduction and elaboration at different levels of linguistic structure to efficiently communicate linguistic meaning.

A number of directions of future research emerge from our study. Firstly, it seems promising to compare our results with those that could be obtained by adopting a dynamic approach to rhythmic units in speech, which accounts for potential (slight) mismatches between vowel onsets and p-centres [51]. The rhythmic units of spoken Bora could thus be defined dynamically by applying heuristic estimations of p-centres as including varying fractions of syllable onsets. Secondly, a better understanding of the limits of intelligibility of drummed Bora remains a desideratum and should be studied by controlled comprehension experiments. Finally, the present study developed a methodology for the analysis of rhythmic structures of drummed speech, which can now be applied to other languages, particularly the several African languages that are known to have developed systems of drummed speech. We thus provide the opportunity for further exciting research on the role of rhythmic structures in drummed and spoken forms of language.

## Supplementary Material

ESM 1

## Supplementary Material

ESM 2

## Supplementary Material

ESM 3-Figure1A_Spoken

## Supplementary Material

ESM 4-Figure1B_Drummed

## Data accessibility

Our data are provided in open access in the documents electronic supplementary material S1.pdf and S2.xlsx (electronic supplementary material, files 1 and 2). These data correspond to measurements carried out on audio files and associated annotations of drummed data from the Bora language documentation online archive at https://hdl.handle.net/1839/00-0000-0000-000C-1B20-E@view (see detail in electronic supplementary material, S1-a).
