# Supplementary material for: Reducing language to rhythm: Amazonian Bora drummed language exploits speech rhythm for long-distance communication
Source: R Soc Open Sci. 2018 Apr 25;5(4):170354. doi: 10.1098/rsos.170354 (PMC5936885; doi:10.1098/rsos.170354)
Supplement: ESM 1 [file rsos170354supp1.pdf]

Electronic Supplementary Material File 1 (ESM1)
for *Reducing language to rhythm: Amazonian Bora drummed speech exploits linguistic rhythm for long-distance communication*
published in the Royal Society Open Science

by Frank Seifart*, Julien Meyer*, Sven Grawunder, Laure Dentel

* F.S. and J.M. contributed equally to this work

Contents
ESM1-a: Data sources
ESM1-b: Distribution of inter-beat durations (IBDs)
ESM1-c: Rhythmic and tone patterns in proper names
ESM1-d: Noun vs. verb marker

ESM1-a: Data sources
Details of all measurements on which statistical analysis was performed out are provided in the document ESM2.xlsx (Electronic Supplementary File 2). These measurements were carried out on audio files and associated annotations of drummed data from the Bora language documentation online archive at https://hdl.handle.net/1839/00-0000-0000-000C-1B20-E@view. Within this collection, some files are accessible without any restrictions also for anonymous users, e.g. https://hdl.handle.net/1839/00-0000-0000-000C-DC56-2@view and https://hdl.handle.net/1839/00-0000-0000-000C-DC94-8@view, which both illustrate messages from Figure 3 (main text). Access to other files in this collection requires registration. Spoken Bora audio files and associated annotations were selected from elicited spoken Bora data archived under https://hdl.handle.net/1839/00-0000-0000-0008-38E9-8@view. These data include spoken pronunciations of phrases from manguaré message and names contained in them (file names mang_habl, mang_habl_3) and elicitation of names of animal species, in singular, dual and plural forms (aves_bora_01, aves_bora_02, aves_bora_03, aves_bora_04, aves_bora_05, mamif_mm_ctxt, mamif_mm_solo, reptiles_01, reptiles_02, reptiles_03, reptiles_04, reptiles_05, reptiles_06, reptiles_07).

ESM1-b1: Details of distribution of inter-beat durations (IBDs)
The following tables provide details on the distribution of inter-beat durations (IBDs) that are summarized in Figure 5 (main text) and Figure ESM-1-1 (below).

Table ESM1-1: Syllable durations in drummed Bora (VV = long vowel)

Drummer	Syllable types	V	VV	CV	VC	CVC	CVV
1	Nb items	1022	128	3873	164	909	1357
1	Mean (ms)	205.6	263.5	177.0	267.6	258	226.9
1	Std (ms)	47.5	41.6	61.4	41.8	49.9	64.9
2	Nb items	236	14	997	29	241	369
2	Mean (ms)	198.5	250.6	174.9	253.4	249.5	200.7
2	Std (ms)	31.1	23.5	43.7	60.6	39.8	59.3

Table ESM1-2: V-to-V durations in drummed Bora (VV = long vowel)

Drummer	V-to-V types	V	VV	VC	VVC	VCC
1	Nb items	483	0	4414	1489	1073
1	Mean (ms)	110.5	X	190.9	229.6	259.5
1	Std (ms)	59.7	X	54.4	64.4	48.9
2	Nb items	60	0	1173	383	270
2	Mean (ms)	141.8	X	181.3	202.5	249.9
2	Std (ms)	38.4	X	41.9	59.3	42.4

Table ESM1-3: V-to-V durations in spoken Bora (VV = long vowel)

Speaker	V-to-V types	V	VV	VC	VVC	VCC
1	Nb items	140	12	642	320	164
1	Mean (ms)	164.6	X	241.5	402.9	363.5
1	Std (ms)	52.8	X	75.8	104.8	103
2	Nb items	67	20	396	159	89
2	Mean (ms)	140.3	X	213.2	324.4	370.1
2	Std (ms)	42.7	X	55.5	68.3	82.5

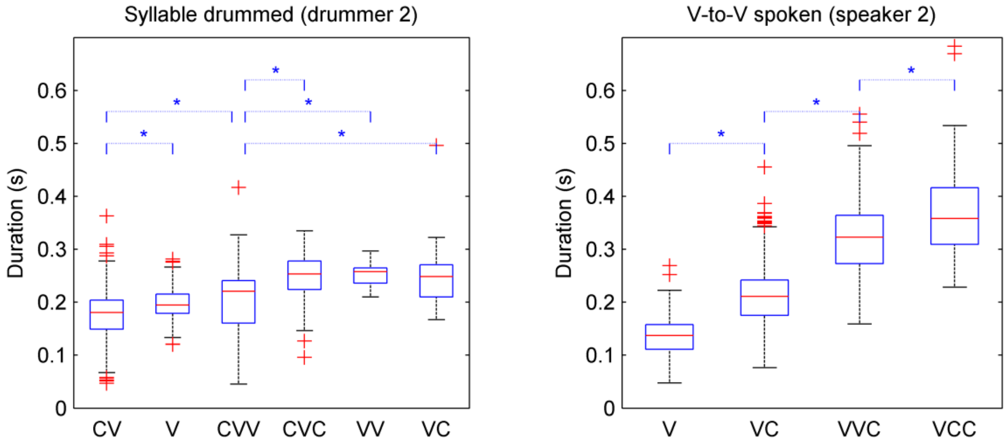

Figure ESM1-1: Left: Drummed IBDs as a function of syllable types for drummer 2. Right: Interval durations of spoken V-to-V types for speaker 2. As explained in the main text, we find here the same distribution as for speaker 1 and drummers

ESM1-b2: Details of the statistical tests associated to the linear models

Table ESM1-4 Results of the multiple comparisons between categories of SYLLABLETYPE (V, CV, CVV, CVC, VV, VC) for each DRUMMER (1 and 2). The first column of the Table indicates the hypothesis of the test for each line. For example, in the first line we compare CV to CVC for drummer 1. The column 'estimate' contains the difference between coefficients of the regression. For example, CVC is shorter than CV by 0.081 ms (first line). The next column gives the standard errors. The column 'z value' gives the value of the statistical test. The column 'value Pr(>|z|)' gives the p-value of the test. The last three columns report inconsistencies internal to the model (Inconsist.), inconsistencies regarding the 'vowel length only' and the 'mora' hypotheses (marked by a '0').

hypothesis	Estimate	Std. Error	z value	Pr(>|z|)	Inconsist.	V vs.VV	Mora
CV - CVC | 1	-0.081	0.0021	-39.2002	**<0.001**		0	
CV - CVV | 1	-0.0499	0.0018	-28.1959	**<0.001**			
CV - V | 1	-0.0286	0.002	-14.5256	**<0.001**	Duration	0	
CV - VC | 1	-0.0906	0.0045	-20.2598	**<0.001**		0	
CV - VV | 1	-0.0865	0.005	-17.1662	**<0.001**			
CVC - CVV| 1	0.0311	0.0024	12.9555	**<0.001**		0	0
CVC - V | 1	0.0524	0.0026	20.4846	**<0.001**		0	
CVC - VC | 1	-0.0096	0.0048	-2.0089	0.506			
CVC - VV | 1	-0.0055	0.0053	-1.032	0.988		0	
CVV - V | 1	0.0212	0.0023	9.1421	**<0.001**	#Drummer2		
CVV - VC | 1	-0.0407	0.0046	-8.7784	**<0.001**	Duration	0	0
CVV - VV | 1	-0.0366	0.0052	-7.0591	**<0.001**	Duration	0	0
V - VC | 1	-0.0619	0.0047	-13.1285	**<0.001**		0	
V - VV | 1	-0.0578	0.0053	-10.9999	**<0.001**			
VC - VV | 1	0.0041	0.0066	0.619	0.9998		0	
CV - CVC | 2	-0.0746	0.004	-18.5421	**<0.001**		0	
CV - CVV | 2	-0.0258	0.0034	-7.5404	**<0.001**			
CV - V | 2	-0.0236	0.0041	-5.8126	**<0.001**	Duration	0	
CV - VC | 2	-0.0785	0.0106	-7.4334	**<0.001**		0	
CV - VV | 2	-0.0757	0.0151	-5.0143	**<0.001**			
CVC - CVV| 2	0.0489	0.0046	10.5224	**<0.001**		0	0
CVC - V | 2	0.051	0.0051	9.9388	**<0.001**		0	
CVC - VC | 2	-0.0039	0.011	-0.3527	1			
CVC - VV | 2	-0.001	0.0154	-0.0675	1		0	
CVV - V | 2	0.0022	0.0047	0.4643	1	#Drummer1	0	0
CVV - VC | 2	-0.0528	0.0108	-4.8783	**<0.001**	Duration	0	0
CVV - VV | 2	-0.0499	0.0153	-3.2688	**0.022**	Duration	0	0
V - VC | 2	-0.0549	0.011	-4.9778	**<0.001**		0	
V - VV | 2	-0.0521	0.0154	-3.3763	**0.015**			
VC - VV | 2	0.0028	0.0182	0.156	1		0	

Table ESM1-5 Results of the multiple comparisons between categories of V-TO-VTYPE (V, VC, VVC, VCC) for each DRUMMER (1 and 2).

Hypothesis	Estimate	Std. Error	z value	Pr(>|z|)
V - VC | 1	-0.0804	0.0026	-30.9089	**<0.001**
V - VCC | 1	-0.1489	0.003	-50.0875	**<0.001**
V - VVC | 1	-0.1191	0.0028	-41.9082	**<0.001**
VC - VCC | 1	-0.0685	0.0018	-37.1104	**<0.001**
VC - VVC | 1	-0.0387	0.0016	-23.7953	**<0.001**
VCC - VVC| 1	0.0298	0.0022	13.7349	**<0.001**
V - VC | 2	-0.0395	0.0072	-5.5008	**<0.001**
V - VCC | 2	-0.1081	0.0077	-13.9603	**<0.001**
V - VVC | 2	-0.0607	0.0075	-8.0512	**<0.001**
VC - VCC | 2	-0.0686	0.0037	-18.7318	**<0.001**
VC - VVC | 2	-0.0211	0.0032	-6.6229	**<0.001**
VCC - VVC| 2	0.0475	0.0043	11.0065	**<0.001**

Table ESM1-6 Results of the multiple comparisons between categories of V-TO-VTYPE for each comparison (DRUMMER (1, 2); SPEECHTYPE(D for Drummed, O for Oral).

Hypothesis	Estimate	Std. Error	z value	Pr(>|z|)
V - VC | 1,D	-0.0804	0.0028	-28.4059	**<0.001**
V - VCC | 1,D	-0.1489	0.0032	-46.0314	**<0.001**
V - VVC | 1,D	-0.1191	0.0031	-38.5145	**<0.001**
VC - VCC | 1,D	-0.0685	0.002	-34.1052	**<0.001**
VC - VVC | 1,D	-0.0387	0.0018	-21.8683	**<0.001**
VCC - VVC|1,D	0.0298	0.0024	12.6226	**<0.001**
V - VC | 2,D	-0.0395	0.0078	-5.0554	**<0.001**
V - VCC | 2,D	-0.1081	0.0084	-12.8298	**<0.001**
V - VVC | 2,D	-0.0607	0.0082	-7.3992	**<0.001**
VC - VCC | 2,D	-0.0686	0.004	-17.2149	**<0.001**
VC - VVC | 2,D	-0.0211	0.0035	-6.0866	**<0.001**
VCC - VVC|2,D	0.0475	0.0047	10.1152	**<0.001**
V - VC | 1,O	-0.0769	0.0055	-13.9545	**<0.001**
V - VCC | 1,O	-0.2382	0.0068	-35.0684	**<0.001**
V - VVC | 1,O	-0.1989	0.006	-33.2404	**<0.001**
VC - VCC | 1,O	-0.1614	0.0052	-31.2433	**<0.001**
VC - VVC | 1,O	-0.122	0.004	-30.2009	**<0.001**
VCC -VVC|1,O	0.0394	0.0057	6.945	**<0.001**
V - VC | 2,O	-0.0728	0.0078	-9.339	**<0.001**
V - VCC | 2,O	-0.2298	0.0095	-24.0667	**<0.001**
V - VVC | 2,O	-0.1841	0.0086	-21.4102	**<0.001**
VC - VCC | 2,O	-0.157	0.0069	-22.6665	**<0.001**
VC - VVC | 2,O	-0.1113	0.0055	-20.0751	**<0.001**
VCC -VVC|2,O	0.0457	0.0078	5.8482	**<0.001**

ESM1-c: Rhythmic and tone patterns in proper names

Table ESM1-7 Rhythmic and tone patterns of all trisyllabic proper names contained in our drummed data. Note that phrase-final rhythmic intervals necessarily carry low tone and are necessarily V-intervals. The theoretically possible HH(L) tone pattern did not occur.

	Proper name	Rhythmic pattern	Tone pattern
1.	ʤìíˀʲò	V.VC(.V)	LH(L)
2.	βàíˀʲò		LH(L)
3.	tùmɨ́ʔhɨ̀	VC.VCC(.V)	LH(L)
4.	ʤìkʲáʔpà		LH(L)
5.	nʲùrɨ́ɨ́bè	VC.VVC(.V)	LH(L)
6.	ʤíhkʲàù	VCC.V(.V)	HL(L)
7.	mɨ̀hkóì		LH(L)
8.	néʔnìbà	VCC.VC(.V)	HL(L)
9.	núʔbàhè		HL(L)
10.	bèʔhɨ́kò		LH(L)
11.	ɨ̀hkóʔè		LH(L)
12.	mìhʲnákò		LH(L)
13.	ròʔʣɨ́bà		LH(L)
14.	úútʲàgʷà	VVC.VC(.V)	HL(L)
15.	ììˀʲágʷà		LH(L)
16.	ʤììkʲáʔè		LH(L)
17.	nèèpáhʲù		LH(L)
18.	ììmúúbè	VVC.VVC(.V)	LH(L)

ESM1-d: Durational difference between noun and verb marker

The verb marker (*-ʔíhkʲà/á*) and noun maker (*-úβù/ú*) involve two contrastive vowel-to-vowel intervals. Firstly, they contained VCC interval (*íhkʲ*) vs. a VC interval (*úβ*). Secondly, these markers impose contrasting intervals preceding these intervals, that is the V-interval preceding *-úβù/ú* and the VC-interval preceding and overlapping with *-ʔíhkʲà/á* (C here being *ʔ*, the first segment of *-ʔíhkʲà/á*). For this contrast, we only consider short vowels given the scarcity of long vowels in our data, especially preceding other vowels with no intervening consonants. Furthermore, we analysed durational contrasts for the intervals contained in the markers (*íhkʲ* vs. *úβ*) separately for the phrase-medial tone pattern HH and the phrase-final tone pattern HL, as phrase-final HL realizations were significantly longer than corresponding HH realizations for both markers, probably reflecting phrase-final lengthening. The preceding intervals (V vs. V*ʔ*) overwhelmingly have the tone pattern HL, as this tone pattern is usually required by these markers. This results in three contrastive intervals (i) HL interval contained in markers, (ii) HH interval contained in markers, and (iii) interval preceding markers (only LH) (see Figures ESM1-2, ESM1-3 and ESM1-4, Table ESM1-8).

For the analysis of durational contrasts, we excluded all instances of the verb marker *-ʔíhkʲà* that occur in the "message types" of calling messages (see Figure 3 in main text), i.e. we excluded instances from *ʦà-ʔíhkʲà* 'Come now!' and *ʦíβà-ʔíhkʲà* 'Bring now!'. Given the formulaic nature of these sequences (every calling message begins with this sequence, and every one contains the verb marker in the same position) the presence of the verb marker in these contexts is entirely predictable and not contrastive. We used only data from drummer 1 (see Table ESM1-8), since only this drummer produced a sufficient amount items for all three contrasts in non-formulaic phrases. To analyse the durational contrasts, we applied nonparametric tests since the normality assumption for

a t-test were violated in these sub-samples. Wilcoxon rank sum tests (with continuity correction in R) indicate statistically significant contrasts for all three constellations: LH Interval preceding markers W = 537.5, p-value = 4.268e-05; HL interval contained in markers: W = 537.5, p-value = 4.268e-05; HH interval contained in markers: W = 134, p-value = 4.378e-06.

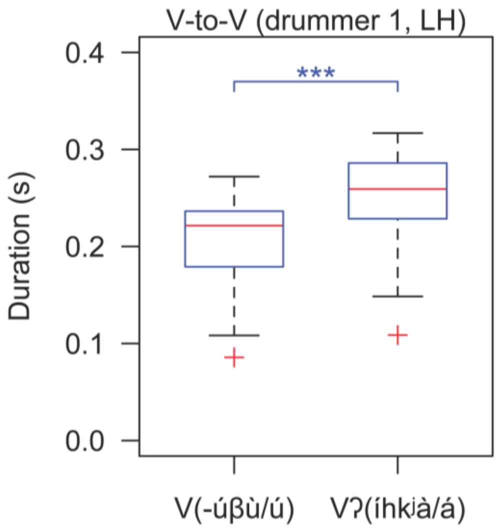

Figure ESM1-2: Durational contrast of intervals preceding verb marker (-*ʔíhkʲà/á*) vs. noun maker (-*úβù/ú*)

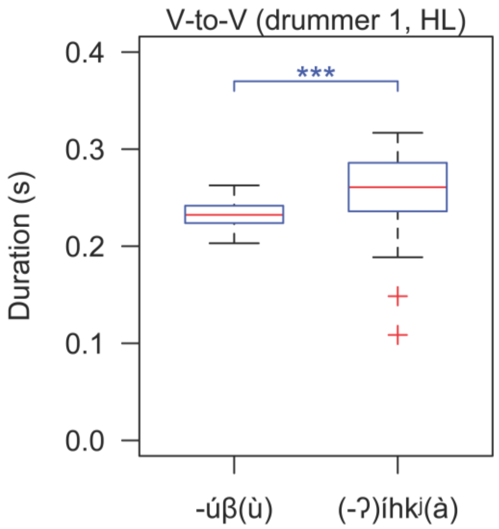

Figure ESM1-3: Durational contrast of intervals contained in verb marker (-*ʔíhkʲà/á*) and noun maker (-*úβù/ú*) with HL tone configuration

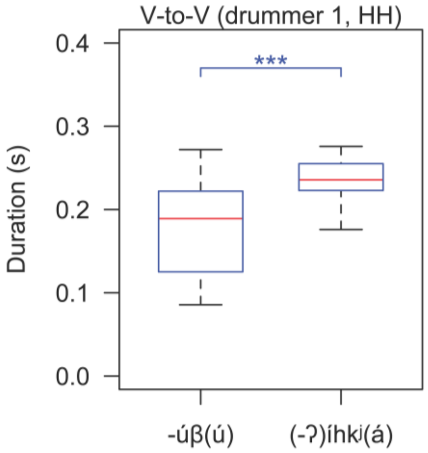

Figure ESM1-4: Durational contrast of intervals contained in verb marker (-*ʔíhkʲà/á*) and noun maker (-*úβù/ú*) with HL tone configuration

Table ESM1-8 V-to-V durations in drummed Bora verb and noun marker

V-to-V types	*íhkʲ* HL	*úβ* HL	*íhkʲ* HH	*úβ* HH	V(-*úβù/ú*)	Vʔ(*íhkʲà/á*)
Nb items	88	26	22	38	64	110
Mean (ms)	256.8	232.0	238.2	183.7	203.3	253.1
Std (ms)	36.6	14.6	23.1	50.9	46.6	35.0
